# Sustained release of metformin through an injectable hydrogel for synergistic macrophage reprogramming and ROS scavenging in the analgesic treatment of osteoarthritis

**DOI:** 10.1080/10717544.2026.2738344

**Published:** 2026-10-29

**Authors:** Jie Xu, Yuhao Chen, Xuan Zhao, Huiyun Li, Honggui Chen, Xiang Ding, Yang Cui, Tingjian Wang, Ning Wang, Caifeng Deng, Guanghua Lei, Chao Zeng, Hui Li

**Affiliations:** a Department of Orthopaedics, Xiangya Hospital, Central South University, Changsha, China; b Key Laboratory of Aging-related Bone and Joint Diseases Prevention and Treatment, Ministry of Education, Xiangya Hospital, Central South University, Changsha, China; c Hunan Key Laboratory of Joint Degeneration and Injury, Xiangya Hospital, Central South University, Changsha, China; d Department of Neurosurgery, Xiangya Hospital, Central South University, Changsha, China; e National Clinical Research Center for Geriatric Disorders, Xiangya Hospital, Central South University, Changsha, China

**Keywords:** Osteoarthritis, metformin, hydrogels, pain, macrophage

## Abstract

Osteoarthritis (OA), a prevalent joint disorder affecting millions worldwide, is primarily characterized by chronic pain driven by synovial M1-polarized macrophages, which perpetuate inflammation and generate excessive reactive oxygen species (ROS). However, existing pharmacological treatments offer limited long-term efficacy. Metformin, a known modulator of M1 macrophage polarization and ROS production, has demonstrated efficacy in alleviating OA pain via oral administration. Yet, its therapeutic translation is hindered by poor joint tissue bioavailability. Intra-articular (IA) delivery offers a clinically attractive solution, achieving high local drug concentrations while minimizing systemic exposure, but conventional IA formulations are rapidly cleared from the joint space, restricting their efficacy. We designed an injectable hydrogel based on dopamine (DA) and carboxyl-terminated Pluronic F127 (F127) and crosslinked the hydrogel network via oxidative coupling between catechol groups using an H_2_O_2_/horseradish peroxidase (HRP) system (termed ‘F127-DA-HRP’) to deliver metformin (termed ‘Met-F127-DA-HRP’) intra-articularly in a sustained manner. The Met-F127-DA-HRP hydrogel scavenges ROS through its catechol groups and sustained metformin release, while also promoting M1-to-M2 macrophage repolarization and suppressing inflammatory cytokines. Importantly, a single IA injection of Met-F127-DA-HRP hydrogel maintained 2-fold higher metformin concentrations in joint tissues compared to Met-F127 hydrogel, with sustained retention for up to 30 days. In monosodium iodoacetate (MIA)-induced OA, IA injection of Met-F127-DA-HRP hydrogel successfully relieved OA pain and ameliorated synovitis with long-lasting effects. Collectively, this work introduces a potent therapeutic strategy for the sustained management of OA pain by utilizing an injectable hydrogel to prolong the IA residency of metformin, offering long-acting IA analgesia through macrophage-directed immunomodulation.

## Graphical abstract

**Figure:**
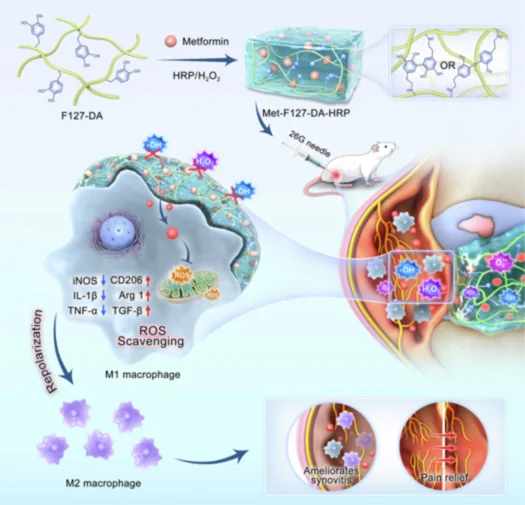

## 1. Introduction

Knee osteoarthritis (OA) is a highly prevalent condition, affecting an estimated 600 million people globally as of 2021 (Brauer et al. 2024), and its incidence and prevalence are increasing. Joint pain from OA is a leading cause of people seeking medical treatment and is a major source of chronic disability (Hunter and Bierma-Zeinstra 2019). The main goal of medical therapy for managing OA is to minimize pain and optimize function (Bijlsma et al. 2011). Although systemic analgesics-including non-steroidal anti-inflammatory drugs (NSAIDs), paracetamol, and opioids-are commonly used to manage OA pain, their association with adverse events (e.g. cardiovascular and gastrointestinal complications, addiction) presents substantial clinical limitations (Hunter et al. 2014). Current pharmacological interventions for OA provide only temporary symptomatic alleviation and lack the capacity for sustained, safe analgesia. Therefore, developing novel therapeutic strategies capable of providing sustained relief from chronic OA pain represents a critical unmet medical need.

Metformin is a first-line, orally administered antihyperglycemic agent (Flory and Lipska 2019; Zaccardi et al. 2020). Beyond glycemic control, accumulating evidence demonstrates its broad pharmacological effects, including the regulation of macrophage polarization (Yao et al. 2024; Wang et al. 2025; Jafarzadeh et al. 2025) and the suppression of reactive oxygen species (ROS) production (Feng et al. 2022; Yang et al. 2025). In OA, synovitis driven by M1-polarized macrophages is a major source of ROS (Chen et al. 2019), which promotes inflammation, matrix dysregulation, and cartilage degeneration (Lepetsos et al. 2019; Tudorachi et al. 2021; Jiang et al. 2025). These mechanisms provide a rationale for repurposing metformin in OA, supported by preclinical evidence that oral metformin alleviates OA-related pain through the attenuation of nociceptive signaling and pain severity (Jin et al. 2020; Feng et al. 2021). Moreover, a randomized trial in overweight/obese patients with symptomatic knee OA demonstrated that oral metformin provided moderate pain relief (Pan et al. 2025). Collectively, these findings support the potential of metformin as a disease-modifying and analgesic agent in OA, particularly through the attenuation of synovial inflammation and oxidative stress.

However, as a systemic drug, metformin has relatively low concentrations in joint tissues, and its pharmacokinetics make it difficult to sustain joint protection (Abbasi et al. 2024). For direct-acting therapeutics, intra-articular (IA) administration is generally preferred in severe joint disorders with intense pain, as it facilitates the distribution of high therapeutic drug concentrations throughout the joint capsule (Jones et al. 2019; You et al. 2025). Unfortunately, metformin suffers from rapid clearance by synovial fluid owing to its low molecular weight (Evans et al. 2014; Na et al. 2021), which often requires multiple IA injections (Jin et al. 2020). Therefore, enhancing metformin's intra-articular retention and bioavailability is critical for effective OA therapy.

In recent years, stimuli-responsive hydrogels have attracted much attention in biomedicine due to their capacity to modulate drug release in response to specific environmental cues (Laurano et al. 2021; Neumann et al. 2023; Yu et al. 2024). For instance, thermosensitive hydrogels that undergo a sol-gel transition near physiological temperature form in situ depots upon injection, thereby prolonging drug retention and enabling sustained release (Koland et al. 2022). The stability and biodegradability of hydrogels play a crucial role in controlling the rate of drug release and enhancing treatment efficacy. Moreover, their higher water content imparts lubricating and soothing properties to inflamed tissue, mimicking the native synovial environment (Li and Mooney, 2016). Pluronic F-127 (F127) – a widely used poly(ethylene oxide)-poly(propylene oxide)-poly(ethylene oxide) copolymer (PEO-PPO-PEO) – is the most widely used copolymer for applications in controlled release systems, valued for its low immunogenicity and toxicity (Russo and Villa 2019). However, F127 hydrogel exhibits limited drug release duration due to rapid dissociation in aqueous medium (Li and Mooney, 2016). The unstable structure and poor mechanical strength of F127 hydrogel are also limitations for in vivo application (Li et al. 2020). To address these limitations, covalent cross-linking strategies have been proposed to enhance the stability of temperature-responsive systems after initial gelation (Yu et al. 2011). Dopamine (DA), a melanin-like compound, contains redox-active catechol groups that enable oxidative crosslinking of 3D hydrogels (Liu et al. 2014; Huang et al. 2025). Moreover, DA has emerged as a promising free radical scavenger owing to its catechol-mediated antioxidant activity (Zhang et al. 2025). Therefore, multifunctional hydrogels based on these biomaterials, which are compatible with various therapeutic requirements, are expected to serve as higher-quality delivery systems for OA treatment.

In the present study, we synthesized a two-component injectable hydrogel with excellent stability, rapid gelling process, ROS-scavenging capacity, and long-term sustained drug release based on carboxyl-terminated F127 and the amino groups of DA. The resulting hydrogel was loaded with metformin (Met) and subsequently crosslinked using horseradish peroxidase (HRP) and hydrogen peroxide (H_2_O_2_), yielding the composite denoted as Met-F127-DA-HRP. This process generated a microporous scaffold exhibiting stability in aqueous media, superior temperature sensitivity, and enhanced mechanical performance compared to single-network hydrogels. The Met-F127-DA-HRP hydrogel demonstrated good biocompatibility and the ability to drive macrophage polarization from the M1 to M2 phenotype, scavenge intracellular ROS, and exert anti-inflammatory effects. Furthermore, a single IA injection of Met-F127-DA-HRP hydrogel maintained 2-fold higher metformin concentrations in joint tissues compared to Met-F127 hydrogel, with sustained retention for up to 30 days. In monosodium iodoacetate (MIA)-induced rat models, a single IA Met-F127-DA-HRP hydrogel injection significantly increased analgesic efficacy and strongly promoted the transition from the M1 to M2 phenotype. Collectively, these results indicate that the Met-F127-DA-HRP hydrogel effectively alleviates OA pain, suppresses synovial inflammation, and mitigates early OA progression with long-lasting effects.

## 2. Experimental methods

### 2.1. Materials

Dopamine hydrochloride (purity > 98%) and H_2_O_2_ were purchased from Aladdin Bio-Chem Technology Co., Ltd (Shanghai, China). Pluronic F127 (F127) and HRP were obtained from Sigma-Aldrich. Dichloromethane, triethylamine, 4-nitrophenyl chloroformate, anhydrous ethanol, N, N-dimethylformamide, chloroform, acetonitrile, acetate, and methanol were purchased from Sinopharm Chemical Reagent Co., Ltd. HRP, H_2_O_2_, CDCl_3_, and NaCl were purchased from Aladdin Bio-Chem Technology Co., Ltd. (Shanghai, China). The RAW264.7 cells were purchased from the Shanghai Cell Bank of the Chinese Academy of Sciences.

### 2.2. Preparation and characterization of thermosensitive F127-DA-HRP hydrogel

The F127-DA-HRP hydrogel was synthesized following a previously reported protocol with modifications (Meredith et al. 2014). First, 10g F127 was dissolved in 60 mL dichloromethane with 245.5 μL triethylamine (TEA). The mixture was added dropwise into 10 mL dichloromethane dissolved with 0.9675 g 4-nitrophenyl chloroformate and stirred at room temperature under N_2_. By performing extraction with saturated NaCl and concentrating with cold anhydrous ethanol, we obtained purified F127-NPC. For dopamine conjugation, dopamine hydrochloride (1.00  mmol) was first dissolved in 4 mL N, N-dimethylformamide under an N₂ atmosphere, followed by the addition of TEA (324 μL) after 20 min of stirring. After 20 min of reaction, the F127-NPC dissolved in chloroform was added dropwise and stirred at room temperature under N_2_. After completion of the reaction, dopamine-grafted F127 (denoted as F127-DA) was obtained via dialysis and lyophilization.

To prepare metformin-loaded hydrogels, F127-DA conjugate lyophilized powder was dissolved in a metformin solution prepared in phosphate-buffered saline (PBS, pH 7.4), and the mixture was maintained at 4 °C until complete dissolution. Subsequently, HRP and H₂O₂ were sequentially added to the polymer solution to initiate the enzyme-catalyzed crosslinking reaction. The resulting hydrogels, designated as Met-F127-DA-HRP, were stored at 4 °C for subsequent characterization and use. Drug-free hydrogels (F127-DA-HRP) were prepared following the same procedure, with PBS substituted for the metformin solution.

The chemical structures of the F127-NPC and F127-DA hydrogel were characterized using FTIR (670 FTIR, Nicolet, Madison, WI, USA). F127-NPC and F127-DA lyophilized powders were dissolved in CDCl_3_, and ^1^H NMR spectrometry (AvanceIII 400  MHz, Bruker, Fallanden, Switzerland) was performed. The micromorphology, pore size, and interconnectivity of F127, F127-DA-HRP, and Met-F127-DA-HRP hydrogel were characterized with a scanning electron microscope (SEM, Hitachi, H-7500, Japan). To evaluate hydrogel degradation of the F127-DA-HRP hydrogel under a physiologically relevant OA microenvironment, preformed hydrogels were lyophilized to determine their initial dry weight (W₁). The dried hydrogels were then incubated in 2 mL of human synovial fluid at 37 °C, with the fluid replaced every 2 days. At designated time points, the hydrogels were retrieved, lyophilized, and their remaining dry weight (W₂) was measured. The relative weight was calculated as: Relative weight (%) = (W₂/W₁) × 100%.

To determine the encapsulation efficiency of metformin, the freshly prepared Met-F127-DA-HRP hydrogel was lyophilized and subsequently reconstituted in PBS to fully release the encapsulated metformin into the solution. The resulting solution was filtered, and the metformin concentration was quantified using a UV–vis spectrometry against a standard calibration curve.

### 2.3. Rheological characterization of the F127-DA-HRP hydrogel

The dynamic rheological properties of F127 and F127-DA-HRP hydrogel were characterized using a Discovery HR-2 hybrid rheometer (AR 2000ex, TA Instrument, USA) equipped with a 12-mm parallel plate geometry and a 25-μm gap. The procedure was conducted as described in the previous report (Xu et al. 2017). Briefly, the oscillatory temperature-sweep study was conducted at 10  rad/s and 1% strain from 10 to 40 °C. Oscillatory frequency sweeps (0.1–100 Hz, 1% strain) and Oscillatory time sweeps (1% strain, 10 Hz over 250 s) for the F127-DA-HRP hydrogel were conducted. All rheological measurements were performed in triplicate.

### 2.4. Tribology test

The lubrication performance of F127-DA-HRP hydrogel was evaluated using a universal materials tester (UMT-3, Bruker, Germany). A stainless-steel disk was utilized as the bottom surface, and an 8-mm-diameter polyethylene ball was utilized as the top surface. The tests were performed under the following conditions: an oscillation amplitude of 4 mm, a frequency of 1 Hz, an applied load of 1 N, and a duration of 600 s. The coefficient of friction (COF) was measured using a linear reciprocating tribometer in PBS at 37 °C.

### 2.5. ROS Scavenging of the F127-DA-HRP hydrogel

Using a reported method (Lee et al. 2020), the ROS-scavenging capacity of the F127-DA-HRP hydrogel was evaluated in the presence and absence of a peroxy radical-generating reagent. The F127-DA-HRP hydrogel was incubated with PBS, 600 mM peroxy radical-generating reagent AAPH, 20 mM H_2_O_2_, or 5 mM NaOCl for 1 h. Reaction kinetics were tracked by monitoring absorbance at 375 nm with a microplate reader (MultiskanMK3, Thermo, USA) at predetermined time intervals. Subsequently, the microstructure, pore size, and connectivity of the oxidized F127-DA-HRP hydrogel were examined by SEM.

### 2.6. Drug release from the F127-DA-HRP hydrogel

The release profile of metformin from the F127-DA-HRP hydrogel was quantified by UV–vis spectrophotometry (Metash, China). In brief, the Met-F127-DA-HRP hydrogel was incubated at 37 °C. Thereafter, 10 mL of release medium (including pH 7.4 PBS (0.01 M) and 600 mM AAPH) was carefully layered onto the Met-F127-DA-HRP hydrogel surface without disturbing the matrix. The system was then subjected to continuous agitation at 120 rpm/min in a shaker incubator. The release medium (20 µL) was collected for UV-vis spectrometry and replaced with a fresh release medium.

To explore the relationship among ROS intensity, structural changes, and drug release behavior of F127-DA-HRP hydrogels, the above procedure was repeated using release media containing graded concentrations of AAPH (0, 100, 300, and 600 mM). Concurrently, hydrogel samples exposed to AAPH were retrieved at 24 and 72 h, freeze-dried, and examined by SEM. The pore diameters were then measured from the SEM images using ImageJ software.

### 2.7. *In vitro* biocompatibility assay

The cytocompatibility evaluation of F127, F127-DA-HRP hydrogel, and metformin was performed using the Cell Counting Kit-8 (CCK-8) assay. RAW264.7 cells were seeded in 96-well plates and maintained in Dulbecco's Modified Eagle Medium (DMEM) supplemented with 10% fetal bovine serum (FBS) at 37 °C. After 24 h, the fresh medium containing F127 hydrogel, F127-DA-HRP hydrogel, or metformin was added. After 24, 48, or 72 h of incubation, the cells were washed twice with PBS and incubated with CCK-8 reagent (10 µL/well), followed by absorbance measurement using a microplate reader. RAW264.7 cells were inoculated with an initial density of 5 × 10^4^ per well in a 24-well plate. Then, 10 µL of F127 and F127-DA-HRP hydrogel was added and incubated for 48 and 72 h. Next, 20 µL live/dead staining solution was added for 30 min, protected from light. Fluorescence imaging was performed with a DM6 B automated microscope (Leica Microsystems, Germany).

### 2.8. Detection of intracellular ROS production

To evaluate the antioxidative protective efficacy of F127-DA-HRP hydrogel against extensive oxidative injury, the intracellular ROS content was measured using a fluorescence dye, 2ʹ,7ʹ-dichlorodihydrofluorescein diacetate (DCFH-DA) (Kou et al. 2019). Cells were injured by treatment with 200 µM H_2_O_2_, and then incubated with metformin, the F127-DA-HRP hydrogel, or the Met-F127-DA-HRP hydrogel. Cells were suspended in 10 µM DCFH-DA. The cells were imaged under a DM6 B fluorescence microscope.

### 2.9. Quantification of pro-inflammatory and anti-inflammatory cytokines

The expression of anti- and pro-inflammatory mediators in hydrogel-treated RAW264.7 cells was evaluated via quantitative Real-time polymerase chain reaction (qRT-PCR) and enzyme-linked immunosorbent assay (ELISA). For qRT-PCR, total RNA was extracted from cells using TRIzol reagent. Complementary DNA (cDNA) was then synthesized with the PrimeScript RT reagent kit. Real-time PCR amplification was carried out using a SYBR Green detection system and gene-specific primers targeting matrix-degrading enzymes. All primer sequences are provided in Table S2. For ELISA, following treatment, cell culture supernatants were collected and processed. Subsequently, according to the manufacturer's protocol, interleukin (IL)-1β, IL-6, tumor necrosis factor α (TNF-α), and TGF-β in the processed supernatants were quantified using corresponding ELISA kits (R&D Systems, USA).

### 2.10. Preparation of macrophage-conditioned medium and chondrocyte treatment

To evaluate the paracrine effects of hydrogel-treated macrophages on chondrocyte function (Li et al. 2024), RAW264.7 macrophages were seeded in 6-well plates and stimulated with 100 ng/mL LPS for 24 h to establish an M1 phenotype. The M1 macrophages were treated with F127-DA-HRP hydrogel, Met, and Met-F127-DA-HRP hydrogel for 24 h. The culture supernatants (conditioned media, CM) were harvested and added to primary mouse chondrocytes at a concentration of 50% (v/v) for 48 h. Following incubation, chondrocyte gene expression was analyzed by qRT-PCR, focusing on anabolic markers (*Col2a1*, *Acan*) and catabolic markers (*MMP13*, *ADAMTS5*).

### 2.11. Animals

Male Sprague–Dawley (SD) rats (200 ± 20 g, 8 weeks old) were purchased from Hunan Slack Jingda Experimental Animal Co., Ltd. (Hunan, China). All animals were housed in specific pathogen-free cages at 22 °C ± 2 °C on a 12 h light/dark cycle and were provided with a standard diet. All animal experiments were performed between September 2024 and May 2025.

### 2.12. IA pharmacokinetics testing

Quantitative analysis of metformin retention in OA joints at different time points was conducted using a validated high-performance liquid chromatography (HPLC) method modified from previous reports (Hale et al. 2002). Following IA injection of metformin solution, F127 hydrogel, or F127-DA-HRP hydrogel, synovial fluid and joint tissues were collected from MIA-induced rats on days 0, 14, and 30 post-euthanasia. The joint tissue fragments were homogenized and combined with synovial fluid, as well as deproteinized with acetonitrile, and then centrifuged at 10,000 rpm for 5 min (Upadhyay et al. 2014). 100 μL supernatant was collected, mixed with 100 μL of an acetonitrile internal standard solution (ranitidine hydrochloride, 50 μg/mL), and subsequently vortexed and centrifuged. The analyses were conducted on an Agilent 1260 Infinity II. Separation was performed using a chromatography column C18 (250 × 4.6 mm i.d., 5 µm). Gradient elution was utilized for analysis, 10 mmol/L ammonium acetate (pH 6.8, adjusted with ammonia): methanol (95:5) to ammonium acetate-methanol (80:20) in 25 min. The flow rate was maintained at 1  mL/min, with a column temperature of 30 °C and detection at 233 nm.

### 2.13. Retention time of the F127-DA-HRP hydrogel in an OA joint

The retention time of the F127-DA-HRP hydrogel was evaluated by IA injection in MIA rats (Evans et al. 2014). Briefly, the F127 and F127-DA-HRP hydrogel were loaded with the fluorescence dye Alexa Fluor 750 (AF_750_). After the IA injection of the hydrogel, each fluorescence spectrum in the MIA rats was scanned using a Caliper IVIS Lumina III In Vivo Imaging System (PerkinElmer, Waltham, MA) at designated time points (days 0, 1, 2, 7, 14, and 30). After the final imaging session, the rats were euthanized for subsequent tissue analysis.

### 2.14. Animal experiments and metformin administration

Rats were anesthetized with isoflurane (2% in O_2_) and given a single IA injection of 3 mg MIA (Sigma-Aldrich, UK) in sterile 0.9% saline (Nwosu et al. 2016). Non-osteoarthritic (Sham) rats were utilized as controls. Rats with OA were randomized into 6 groups (*n* = 5): MIA, F127 hydrogel, Met-F127 hydrogel, Met, F127-DA-HRP hydrogel, and Met-F127-DA-HRP hydrogel. The sample size was determined by the investigators according to previous experimental experience. A single 30 µL dose of the matching formulation was delivered via IA injection for all groups with a 31-gauge (31 G) insulin syringe needle; metformin-treated groups were administered at a dose of 0.6 mmol/kg (Jin et al. 2020). After 3 weeks of treatment, rats were deeply anesthetized with sodium pentobarbital (150  mg/kg, i.p.) and euthanized at the experimental endpoint.

### 2.15. Behavioral assessment

Mechanical nociception in rats was assessed using an electronic von Frey apparatus (IITC Life Science Inc.). Rats were individually habituated in transparent enclosures on a raised mesh floor. Mechanical allodynia was then assessed by applying calibrated von Frey filaments to the right hind paw; a positive response (paw withdrawal, licking, or shaking) defined the paw withdrawal mechanical threshold (PWMT) as the minimal force (g) required. PWMT was measured at weekly intervals following MIA injection, with group means calculated from individual animal values. Pain-related weight-bearing asymmetry was quantified using a dual-force transducer system (Linton Instrumentation, Norfolk, UK). Following acclimation in a restrainer, the force (g) sustained by each hindlimb over 5 s was averaged across 5 trials. The load on the affected limb was then expressed as a percentage of the total bilateral hindlimb load, serving as a functional pain index. Assessments were performed weekly. Spontaneous behavior was assessed using the Laboratory Animal Behaviour Observation, Registration and Analysis System (LABORAS, Metris, the Netherlands). Briefly, rats were tested simultaneously, each placed on a separate detection platform, for a continuous 15-hour recording session from 6:00 PM to 9:00 AM the following day. The parameters assessed were the rearing frequency and rearing duration. The tests were performed in a blind manner in that the investigator was not aware of the identification of animals or the study groups.

### 2.16. Histology and immunohistochemistry

For histological staining, formalin-fixed rat knee joints were decalcified for 4 weeks. The decalcified joints were subsequently embedded in paraffin and sectioned at a thickness of 4 μm for Hematoxylin-eosin (H&E) and Safranin O/Fast Green (S.O.) staining. Synovitis score was semi-quantified by the number of synovial lining layers (Mapp et al. 2008). For immunohistochemistry (IHC) staining, knee-joint sections were incubated with antigen retrieval buffer (ab208572, Abcam) for 5  min, and then sequentially treated with 3% H_2_O_2_, and blocked with 5% bovine serum albumin (BSA). Primary antibodies anti-nerve growth factor (NGF), (ab52918, 1:250, Abcam), anti-TNF-α (ab220210, 1:100, Abcam), anti-calcitonin gene-related peptide (CGRP) (ab47027, 1:500, Abcam), and tagged secondary antibodies, goat anti-mouse or goat anti-rabbit, were utilized, followed by a DAB chromogen kit (ZSGB-BIO, Beijing, China). For fluorescence immunostaining, primary antibodies, rabbit polyclonal anti-CD68 (ab125212, 1:200, Abcam), anti-inducible nitric oxide synthase (iNOS) (ab210823, 1:100, Abcam), and anti-CD206 (ab64693, 1:500, Abcam) were incubated overnight at 4 °C, and secondary antibody conjugated to Alexa Fluor 488 (Invitrogen) for 50 min. The fluorescent distributions in the knee joints were observed with a DM6 B fluorescence microscope.

### 2.17. Statistical analysis

All data were presented as the mean ± SD. Comparisons between two groups were analyzed by unpaired two-tailed Student's t-test, while multiple groups were assessed by one‑ or two‑way ANOVA followed by Tukey's post hoc test. All analyses were performed with GraphPad Prism 8.0. Differences were considered statistically significant at *p < 0.05, **p < 0.01, ***p < 0.001, and ****p < 0.0001.

## 3. Results

### 3.1. Preparation and characterization of the F127-DA-HRP hydrogel

We prepared the F127-DA-HRP hydrogel as a safe and effective therapeutic platform for OA. First, we utilized methyl 4-nitrophenyl chloroformate as an end-hydroxyl activating agent to synthesize and characterize dopamine-modified F127-DA (Figure S1). The synthesis of dopamine-conjugated F127 was confirmed by the FTIR and ^1^H NMR (Figures S2 and S3). Then, the F127-DA-HRP hydrogel was formed by adding HRP and H_2_O_2_. The H₂O₂/HRP catalytic system enables rapid and tunable gelation under mild conditions, without generating toxic byproducts from hydrogen peroxide decomposition (Lu et al. 2017). Thus, a series of injectable hydrogels was prepared with H_2_O_2_ contents varying from 0.5% to 1% and 2% in the hydrogel precursor, respectively. As shown in Figure S4A–D, the 1% F127-DA-HRP hydrogel exhibited the best cell affinity, as well as appropriate kinetics of gelation and mechanical properties. Therefore, this concentration was selected for subsequent studies.

As shown in Figure 1A, the appearance of F127-DA-HRP was brown (whereas F127 was clear and transparent) owing to the generation of o-quinone by oxidative conversion of the catechol moiety. As expected, the gelation time (31.3 s) and gelation temperature (22.7 °C) of the cold F127-DA-HRP solution were significantly lower than those of the cold F127 solution (53.6 s and 32.8 °C, respectively) (Table S1). The F127-DA-HRP hydrogel exhibited good injectability, as demonstrated by its smooth extrusion through a 26 G insulin syringe needle at room temperature (Figure 1B). In previous clinical reports, thicker syringe needles, such as 18 G (840 µm inner diameter) or 22 G (410 µm inner diameter), have been utilized to deliver hydrogel systems to cells, which may increase the patient's pain response (Xu et al. 2019). The microstructure of the hydrogel presented a loose, honeycomb-like structure, and its cross-sections revealed its porosity (Figure 1C). The F127-DA-HRP hydrogel had a well-interconnected microporous 3D architecture (Figure 1C), indicating that the dopamine was well connected with F127. As shown in Figure S5, the F127-DA-HRP hydrogel exhibited a time-dependent progressive weight loss, reaching 35.82% ± 4.21% at day 7. This sustained degradation profile supports the favorable biodegradability of the hydrogel system within the OA microenvironment. In addition, the Met-F127-DA-HRP hydrogel achieved a high encapsulation efficiency of 99.49% ± 0.30% (Figure S6).

**Figure 1.**
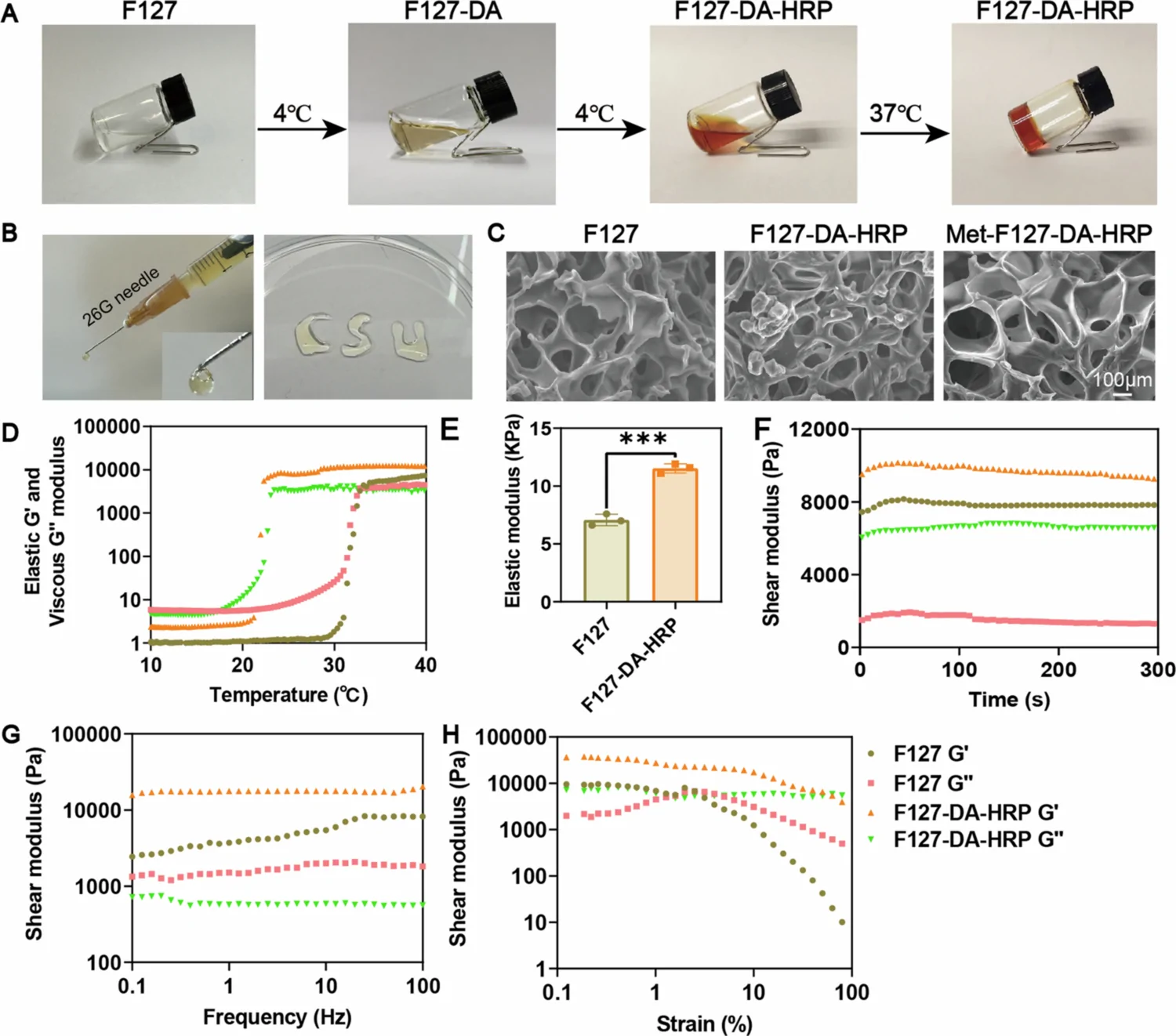
Characterization of F127-DA-HRP hydrogel. (A) Gross view of the F127-DA-HRP hydrogel forming process and reversible gel-sol transitions of the hydrogel triggered by temperature. (B) Injection of F127-DA-HRP hydrogel through a 26 G syringe needle under ambient conditions. (C) SEM image of the F127, F127-DA-HRP, and Met-F127-DA-HRP hydrogel. (D) Storage moduli (G′) and loss moduli (G″) of F127 and F127-DA-HRP hydrogel as a function of temperature from 40 °C to 10 °C. (E) The max elastic moduli of F127 and F127-DA-HRP hydrogel (***p < 0.001, *n* = 3). (F) Oscillatory time sweep experiments for F127 and F127-DA-HRP hydrogel. (G) Oscillatory frequency sweep experiments for F127 and F127-DA-HRP hydrogel. (H) Strain-dependent oscillatory shear rheology of the F127 and F127-DA-HRP hydrogel with a fixed frequency of 10 rad s^−1^.

The formation and physical integrity of the F127-DA-HRP hydrogel were investigated using oscillatory shear rheology. At the gelation temperature of 22 °C, the storage modulus (G′) surpassed the loss modulus (G″), indicating a sol-gel transition (solution state: G′ < G″, gel state: G′ > G″) (Figure 1D). The enzyme-catalyzed crosslinking endowed the F127-DA-HRP hydrogel with significantly enhanced mechanical strength compared to the F127 hydrogel (Figure 1E). Notably, the F127-DA-HRP hydrogel exhibited a 10 °C depression in phase-transition temperature relative to the F127 hydrogel, rendering it more responsive to physiological temperatures. During the initial gelation stage, the hydrogel maintained injectable viscosity while exhibiting rapid curing kinetics. Next, the gelation kinetics and viscoelastic properties of F127-DA-HRP hydrogel were evaluated at physiological temperature (37 °C) using cone-plate rheometry (Figure 1F and G). Oscillating time-sweep measurements revealed that F127-DA-HRP exhibited substantially enhanced viscoelastic moduli (G′ = 10,100 Pa; G″ = 6500 Pa) compared to the control F127 hydrogel (G′ = 7800 Pa; G″ = 1700 Pa) (Figure 1F). These results indicate that F127-DA-HRP can form a stable network structure. Such a network exhibited more elastic than viscous properties and remained intact under shear-stress conditions due to rapidly reversible interactions. In addition, the G′ of the F127-DA-HRP hydrogel remained constant over a wide range of strains, suggesting that it has a highly stable structure under significant deformation (Figure 1G). Furthermore, the F127-DA-HRP hydrogel exhibited high yield stress and strain due to its large surface-area-to-volume ratio, which strengthened interparticle interactions and increased injection resistance while enhancing deformability before yielding (Figure 1H).

Synovial fluid acts as a natural biolubricant for articular cartilage (Seror et al. 2015). In synovitis, altered synovial fluid composition compromises its lubricating capacity (Elsaid et al. 2005). Reciprocating tribometry revealed significantly reduced coefficient of friction (COF) for both the F127 and F127-DA-HRP hydrogel compared to PBS, with COF-time curves and statistical distributions detailed in Figure S7A and B. The functionalization of F127 with dopamine and HRP/H₂O₂ did not compromise its lubricating properties, confirming the retention of effective lubrication in the F127-DA-HRP hydrogel.

### 3.2. ROS scavenging and Cytocompatibility of the Met-F127-DA-HRP hydrogel

During OA progression, elevated levels of ROS are continuously secreted by inflamed synovial tissues. To evaluate its ROS-scavenging capacity, the F127-DA-HRP hydrogel was incubated with PBS or various peroxy radical generators (600 mM AAPH, 20 mM H₂O₂, or 5 mM NaOCl). The antioxidant properties of the hydrogels originated from their unoxidized catechol groups after gelation. As shown in Figure 2A, upon exposure to peroxy radicals (e.g. AAPH) or free radicals (e.g. H₂O₂), the unoxidized catechol groups were oxidized to the corresponding quinone structures, leading to a distinct increase in the absorption peak at 375 nm. Then, the increased release rates of metformin from the hydrogel after incubation with AAPH solution (Figures 2B and S8). Moreover, the oxidation of F127-DA-HRP hydrogel was detected using SEM. Morphological analysis by SEM demonstrated significant microstructural alterations, with hydrogel walls transitioning from a smooth, dense architecture to a porous, irregular morphology post-oxidation (Figure 2C). Notably, all three oxidants markedly increased the average pore diameter of the hydrogel (Figure S9). To further explore the relationship among ROS intensity, structural changes, and drug release behavior of the F127-DA-HRP hydrogel, we conducted release studies using graded AAPH concentrations and examined the corresponding hydrogel microstructure at 24 h and 72 h post-oxidation. As shown in Figure S10A, the cumulative release of metformin increased proportionally with AAPH concentration over 120 h. Concurrently, SEM analysis revealed a marked increase in average pore diameter at 600 mM AAPH compared to the PBS (Figure S10B and C). These findings support an ROS-responsive structural remodeling mechanism underlying the accelerated release of metformin.

**Figure 2.**
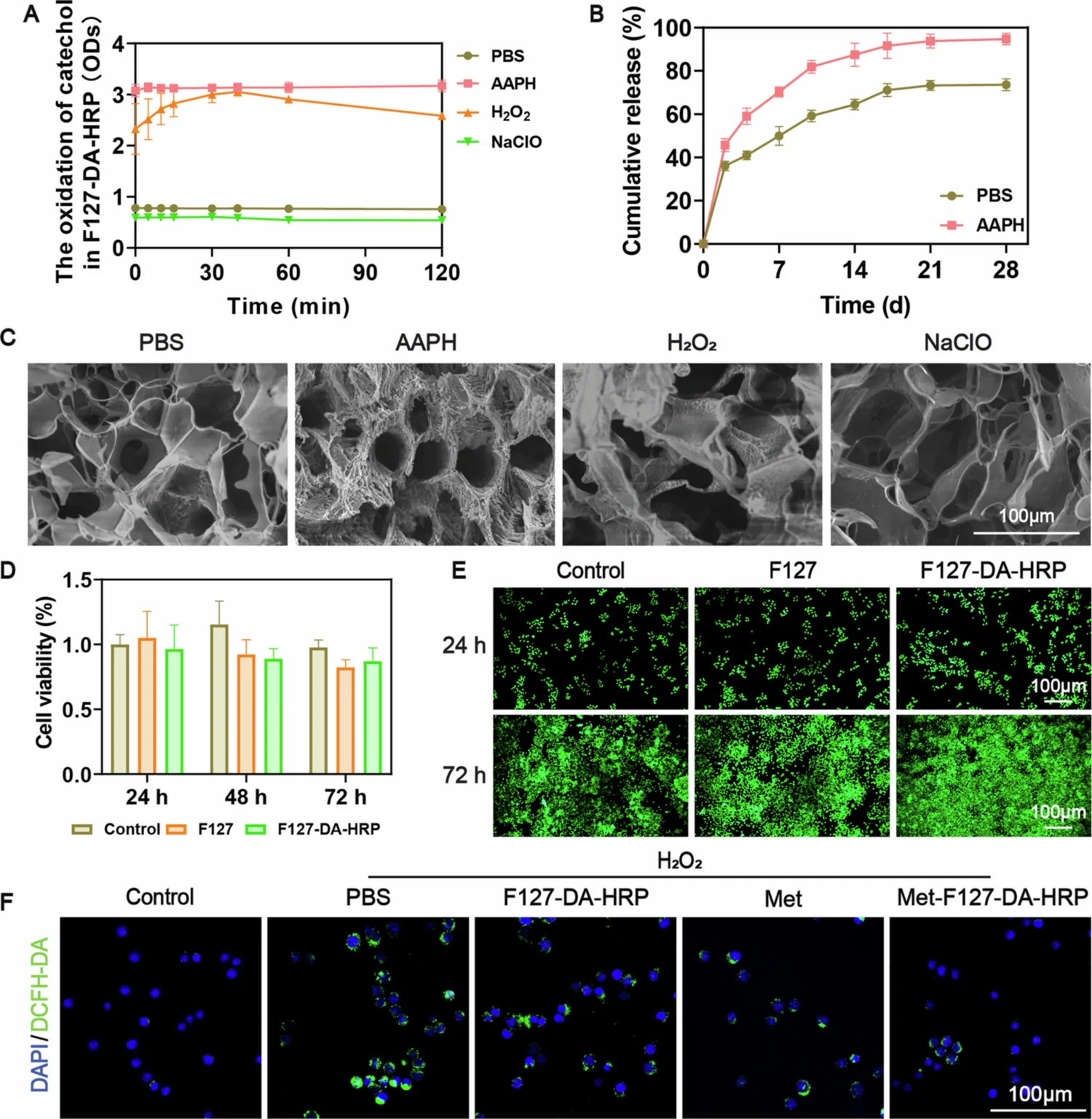
ROS scavenging capability and Cytocompatibility of Met-F127-DA-HRP hydrogel. (A) F127-DA-HRP hydrogel treated with PBS, 600 mM AAPH, 20 mM H_2_O_2_, or 5 mM NaOCl over 1 h. (B) Drug release profiles of the Met-F127-DA-HRP hydrogel in PBS and 600 mM AAPH (37 °C) for 28 d. (C) SEM image of the F127-DA-HRP hydrogel treated with PBS, 600 mM AAPH, 20 mM H_2_O_2_, or 5 mM NaOCl over 1 h. (D) Cell viability (24  h, 48 h, and 72 h) of RAW264.7 incubated with F127 and F127-DA-HRP hydrogel (*n* = 3). (E) Representative images from Live/Dead Viability of F127 and F127-DA-HRP hydrogel after incubating with RAW264.7 cells for 24 h and 72 h. (F) Confocal images of intracellular ROS levels of the RAW264.7 cells treated with Met-F127-DA-HRP hydrogel. All data represent the mean ± S.D.

After confirming catechol-mediated ROS scavenging in F127-DA-HRP, we evaluated the metformin-catechol synergy in Met-F127-DA-HRP; cytotoxicity was first verified via live/dead and CCK-8 assay. As depicted in Figure 2D and E, cell viability remained high in all treatment groups. Correspondingly, live/dead staining revealed no significant reduction in cell survival, consistent with the CCK‑8 results. These results indicate that the F127-DA-HRP hydrogel exhibits favorable biocompatibility. We also verified that metformin treatment had no significant cytotoxic effect on RAW264.7 cells when administered at concentrations ranging from 1 to 20 nM for 24 h (Figure S11).

Having established the excellent biocompatibility of the F127-DA-HRP hydrogel, its capability to remove intracellular ROS was investigated. As shown in Figures 2F and S12A, stimulation with H_2_O_2_ led to a significant increase in the level of ROS. However, M1 macrophages treated with the Met-F127-DA-HRP hydrogel exhibited the most pronounced reduction in intracellular green fluorescence intensity, demonstrating a significantly superior ROS elimination capacity compared to all other treatment groups. Both the F127-DA-HRP hydrogel and metformin effectively reduced the ROS amount. These results demonstrate that the Met-F127-DA-HRP hydrogel directly scavenges extracellular ROS and subsequently suppresses intracellular ROS generation, resulting in synergistic mitigation of oxidative stress.

### 3.3. Met-F127-DA-HRP hydrogel promotes macrophage repolarization from M1 to M2 phenotype

Given the well-documented ability of metformin to regulate the polarization of M1 and M2 macrophages, we further investigated whether the Met-F127-DA-HRP hydrogel could be advantageous in terms of reprogramming the phenotype of macrophages. As shown in Figure 3A and B, and Figure S12B and C, the F127-DA-HRP hydrogel and free metformin exhibited a moderate capacity to decrease the expression of iNOS and increase the expression of CD206 in LPS-stimulated RAW264.7 cells. Notably, the Met-F127-DA-HRP hydrogel was the most efficient in repolarizing M1 macrophages to the M2 phenotype. Moreover, Met-F127-DA-HRP significantly downregulated the transcriptional levels of *iNOS*, *IL-1β*, *IL-6,* and *TNF-α* compared with the F127-DA-HRP hydrogel and free metformin (Figure 3C–F). Correspondingly, it markedly upregulated the expression of M2‑related genes, including *arginase‑1* (*Arg1*), *IL‑4*, *IL‑10*, and *transforming growth factor‑β* (*TGF‑β*), relative to the same controls (Figure 3G–J). Furthermore, it induced the highest transcriptional level of *IL-10* among all experimental groups. While free metformin alone moderately downregulated the expression of IL-1β, IL-6, TNF-α, and upregulated TGF-β (Figure 3K–N), the hydrogel formulation markedly enhanced these effects. These results demonstrated that the Met-F127-DA-HRP hydrogel effectively drives the repolarization of M1 macrophages toward an M2-like phenotype.

**Figure 3.**
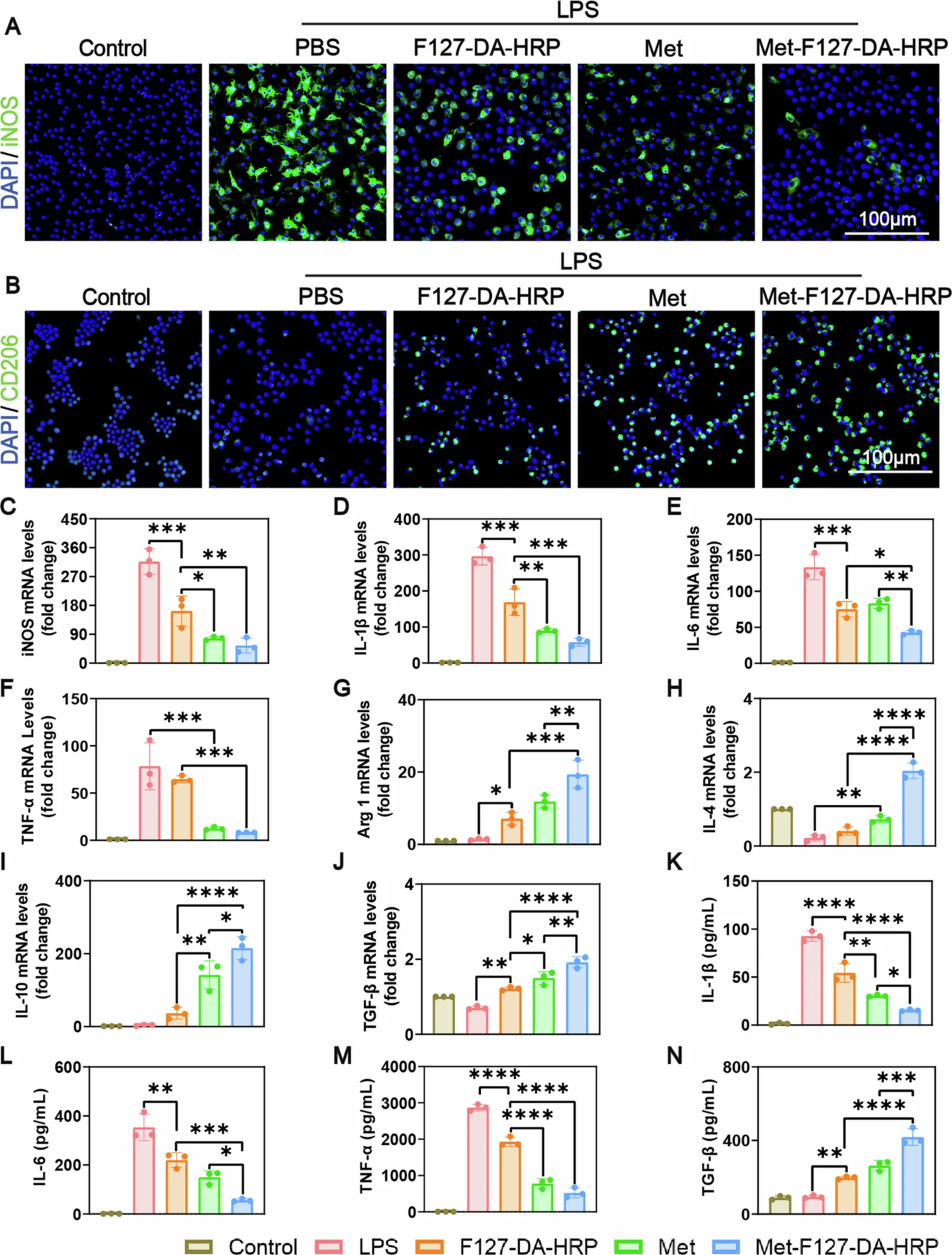
*In vitro* macrophage repolarization from M1 to M2 phenotype by Met-F127-DA-HRP hydrogel. (A) Representative fluorescence images of iNOS staining of macrophages on different samples. (B) Representative fluorescence images of CD206 staining of macrophages on different samples. (C–F) Relative mRNA expression levels of M1- related genes iNOS, IL-1β, IL-6, and TNF-α (*n* = 3). (G–J) Relative mRNA expression levels of M2-related genes Arg-1, IL-10, IL-4 and TGF-β (*n* = 3); (K–N) Protein expression of IL-1β, IL-6, TNF-α and TGF-β (*n* = 3). All data represent the mean ± S.D. *p < 0.05, **p < 0.01, ***p < 0.001, **** *p* < 0.0001.

To evaluate whether Met-F127-DA-HRP hydrogel protects chondrocytes indirectly via regulating macrophage-secreted factors, primary chondrocytes were treated with CM collected from M1 macrophages pre-exposed to various hydrogel formulations. As shown in Figure S13, compared with the CM group, the CM from Met-F127-DA-HRP-treated macrophages significantly upregulated the anabolic genes *Col2a1* and *Acan*, while downregulating the catabolic genes *MMP13* and *ADAMTS5* in chondrocytes.

### 3.4. F127-DA-HRP hydrogel enhances metformin bioavailability and prolongs IA retention in OA joints

Given the excellent ROS scavenging and anti-inflammatory abilities of Met-F127-DA-HRP hydrogel observed *in vitro*, we next evaluated its pharmacokinetic profiles in a MIA-induced rat model of OA. We employed HPLC to detect the retention concentration of metformin in the free Met, Met-F127 hydrogel, and Met-F127-DA-HRP hydrogel after a single IA injection (Figure 4A). After IA administration, metformin concentrations were maintained at homogeneous and comparable levels across the different groups (Figure 4B). Notably, the Met-F127-DA-HRP hydrogel group maintained significantly higher metformin concentrations in joint tissue and synovial fluid mixed samples at both day 14 and day 30 compared to the Met-F127 group (Figure 4C and D). In contrast, free metformin exhibited rapid clearance, with barely detectable levels by day 14. Subsequently, retention times of the F127 and F127-DA-HRP hydrogel in the joint were investigated using fluorescence imaging after IA injection in an MIA rat. For this purpose, both hydrogel formulations were pre-loaded with AF_750_. Compared with the AF_750_-F127 hydrogel, the AF_750_-F127-DA-HRP hydrogel exhibited markedly higher fluorescence intensity in OA joints, which persisted for up to 30 days after injection (Figure 4E–G). These results illustrated that the F127-DA-HRP hydrogel markedly extended drug retention time and increased bioavailability within OA joints.

**Figure 4.**
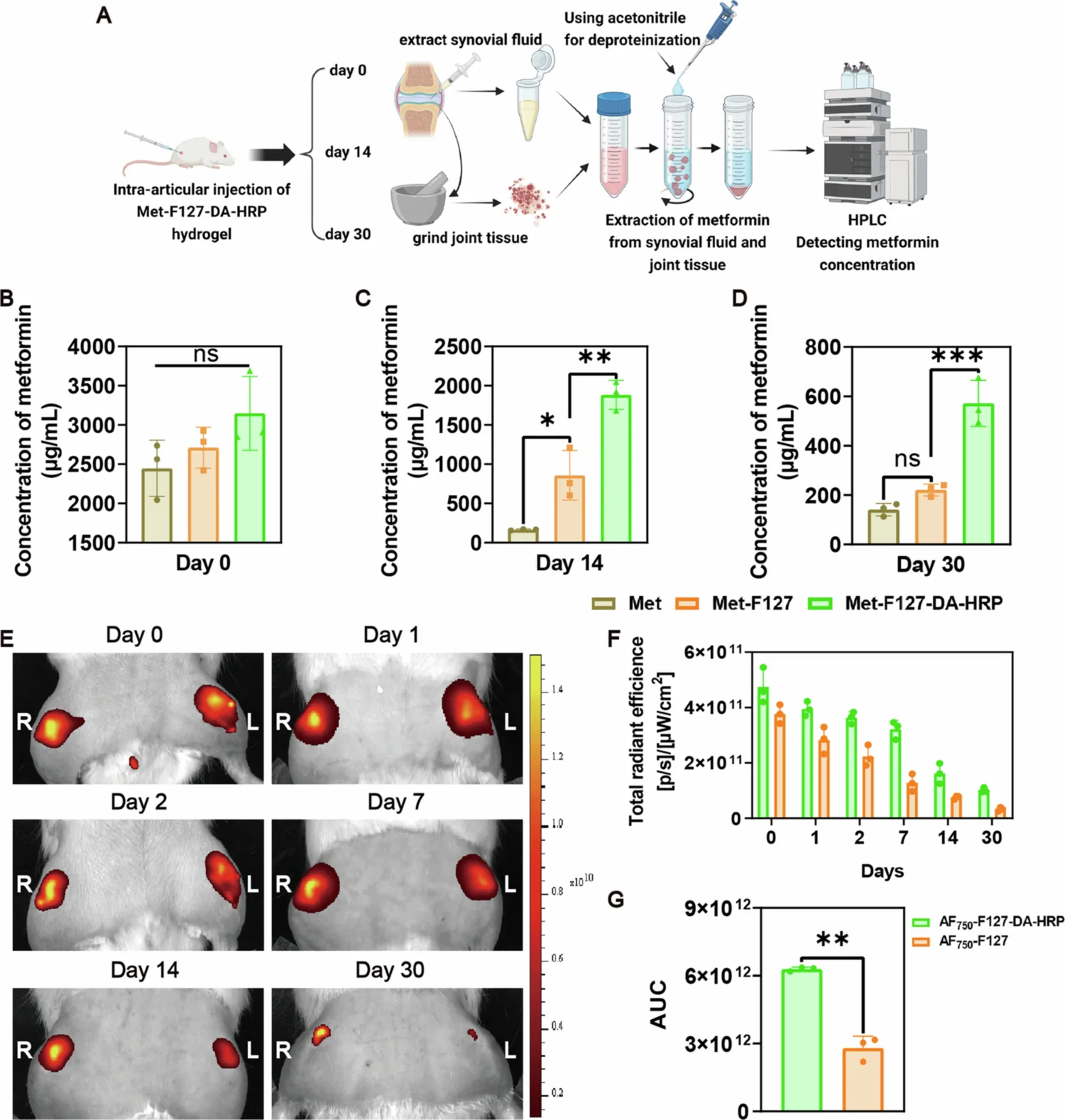
Improved metformin bioavailability and prolonged joint retention by F127-DA-HRP hydrogel. (A) Schematic diagram of detecting the retention of IA metformin concentration by Met-F127-DA-HRP hydrogel using HPLC. (B–D) Quantitative analysis of metformin concentrations in joint tissue and synovium on days 0, 14, and 30 after the injection of free Met, Met-F127 hydrogel, and Met-F127-DA-HRP hydrogel (*n* = 3). (E) Representative fluorescence images of MIA rat knee joints over 30 days after IA injection of AF_750_-F127-DA-HRP (right hind leg) and AF_750_-F127 (left hind leg) hydrogel. (F) Quantitative analysis of time-course fluorescence radiant efficiency within knee joints after a single IA injection of AF_750_-F127 and AF_750_-F127-DA-HRP hydrogel (*n* = 3). (G). Quantitative analysis of the area under the curve based on fluorescence intensity profile in (F) (*n* = 3). All data represent the mean ± S.D. *p < 0.05, **p < 0.01, ***p < 0.001.

### 3.5. Met-F127-DA-HRP hydrogel alleviates pain and suppresses synovial inflammation in MIA-induced OA rats

On the basis that the F127-DA-HRP hydrogel had an extended joint retention time (up to 30 days post-injection), we hypothesized that it may more effectively inhibit OA progression over a longer treatment duration than metformin solution. Therefore, we administered a single dose via IA injection of the F127 hydrogel, F127-DA-HRP hydrogel, Met, Met-F127 hydrogel, and Met-F127-DA-HRP hydrogel into the right knee joint of each rat 1 week after MIA injection (Figure 5A). To evaluate the analgesic efficacy of Met-F127-DA-HRP hydrogel in OA, we conducted a series of behavioral tests to assess pain sensitivity. At 1 week post-modeling, both MIA and treatment groups exhibited significantly lower PWMT and weight-bearing asymmetry in the ipsilateral hindlimb compared to the sham group (Figures 5B and S14A), confirming MIA-induced hyperalgesia. A single injection of metformin only transiently increased PWMT and reduced asymmetry, with effects diminishing by week 2. By contrast, MIA-induced OA rats treated with the Met-F127 and Met-F127-DA-HRP hydrogel had significantly higher PWMT and weight-bearing asymmetry. Notably, IA Met-F127-DA-HRP injection alleviated persistent mechanical allodynia to a much greater extent than Met-F127 (Figures 5C and S14B). We also tested the spontaneous activity of the rat in response to MIA injection and Met-F127-DA-HRP hydrogel treatment. We found that the rearing frequency (Figures 5D and S14C) and rearing duration (Figures 5E and S14D) significantly decreased 4 weeks after MIA injection. Treatment with the Met-F127 and Met-F127-DA-HRP hydrogel significantly reversed the reduced spontaneous activity caused by MIA injection. Remarkably, the rearing duration in the Met-F127-DA-HRP group was longer than that in the Met-F127 group, accompanied by weight-bearing asymmetries closer to those of the sham group. These results indicate that, beyond mitigating mechanically induced pain sensitivity, Met-F127-DA-HRP can restore spontaneous activity suppressed in rats after MIA injection.

**Figure 5.**
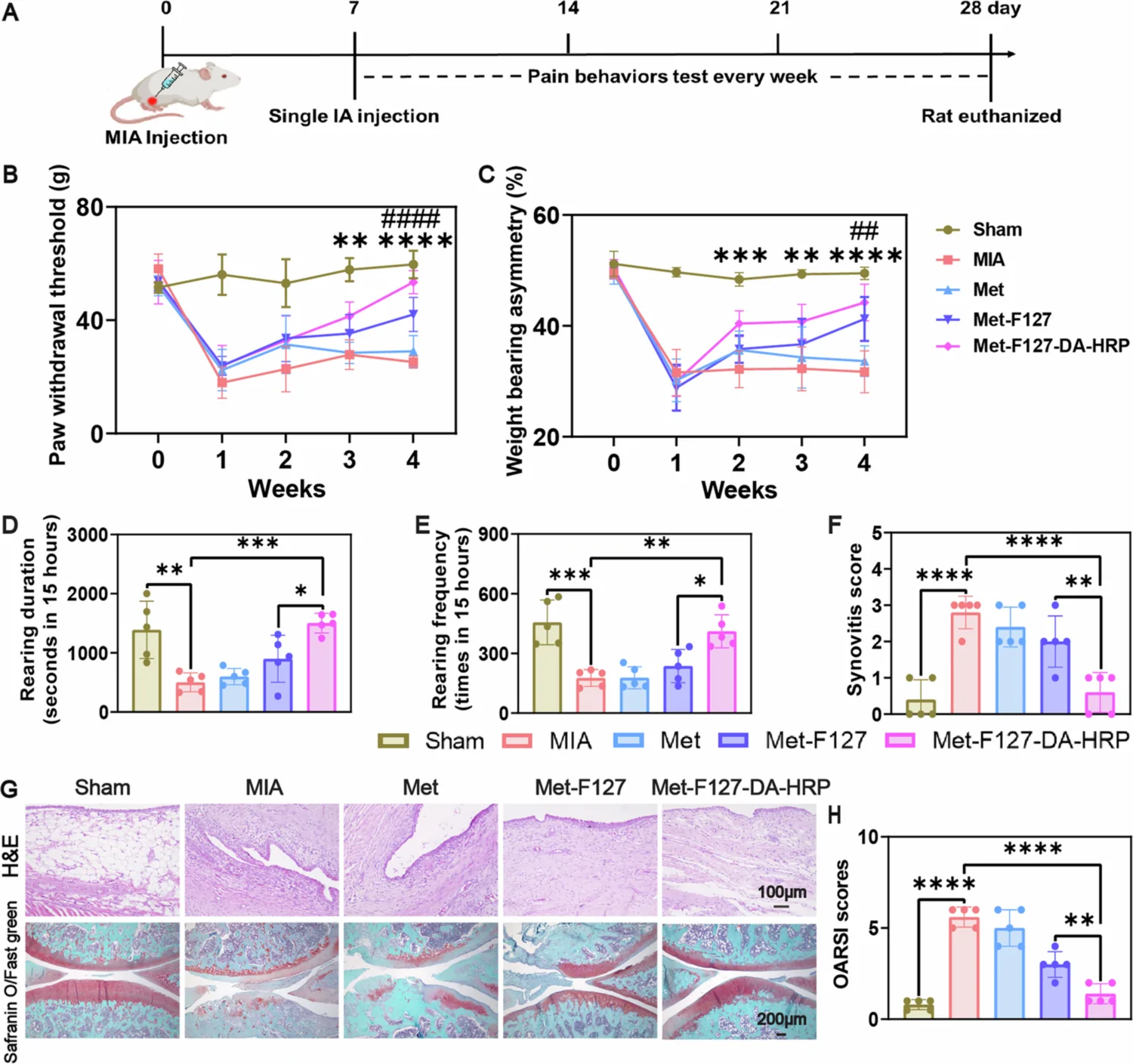
IA injection of Met-F127-DA-HRP hydrogel relieves pain and ameliorates synovial inflammation in MIA rats. (A) Schematic illustration of the study protocol using the MIA rat model. (B) Mechanical sensitivity was measured weekly using von Frey filaments in rats. (C) Changes in weight distribution in rats every week after surgery. *p* values were compared between Met-F127 (#), Met-F127-DA-HRP (*) groups and the MIA group. (D and E) Changes in spontaneous activity, including rearing duration (D) and rearing frequency (E), were evaluated using the LABORAS 4 weeks after MIA injection (*n* = 5). (F) The synovitis scores of samples from the different groups (*n* = 5). (G)The OARSI scores of cartilage samples from different groups (*n* = 5). (H) Representative images of H&E and Safranin O/Fast green-stained sections of knee joints from rats in different groups. All data represent the mean ± S.D. *p < 0.05, ## or **p < 0.01, ***p < 0.001, and #### or ****p < 0.0001.

Synovitis, a key pathological feature of OA, is closely correlated with both pain symptoms and disease progression (Sanchez-Lopez et al. 2022). H&E staining revealed thickening of the synovial lining layer and increased synovitis scores in MIA rats, but not in normal rats (Figures 5F, G, and S14E, G). Notably, the Met-F127-DA-HRP hydrogel treatment group exhibited the most pronounced attenuation of synovial pathology, with markedly reduced synovial hypertrophy, decreased synovial lining cell density, diminished mononuclear cell infiltration, and limited pannus formation. Given the potential use of this material in direct contact with osteoarticular tissues, we examined the integrity of the cartilage surface by S.O. staining. As shown in Figures 5G, H, and S14F, G, in contrast with normal rats, cartilage degeneration was severe in MIA rats, evidenced by markedly increased Osteoarthritis Research Society International (OARSI) scores. The administration of the Met-F127 and Met-F127-DA-HRP hydrogel after MIA injection caused a partial but significant reduction in cartilage degradation. We observed that the F127 and F127-DA-HRP groups had higher integrity and more regular cartilage structure than the MIA group (Figure S14F). These results demonstrated that a single IA injection of Met-F127-DA-HRP hydrogel not only alleviates pain and synovial inflammation but also preserves cartilage structure, mediated through both metformin's therapeutic effects and the hydrogels' biolubricating properties.

Finally, the long-term local toxicity of a single injection of the F127-DA-HRP hydrogel into the knee joint of rats was evaluated using TUNEL staining. No significant apoptotic signal was detected in either the F127 or F127-DA-HRP hydrogel groups (Figure S15A, B), indicating good biosafety of the F127-DA-HRP hydrogel.

### 3.6. Met-F127-DA-HRP hydrogel reversed the phenotypes of M1 macrophage polarization and promoted M2 macrophage polarization in MIA-induced OA rats

Given the evidence that synovial macrophages play an important role in the progression of OA, we further investigated the modulatory effects of Met-F127-DA-HRP hydrogel on macrophage polarization in MIA-induced OA rats (Figures 6A–D and S16A–D). In the MIA rats, a significant elevation in the number of CD68 and iNOS-positive cells was detected. A single injection of metformin slightly reduced CD68-positive and iNOS-positive cell counts, with a low level of CD206 expression. Notably, the Met-F127-DA-HRP hydrogel significantly reduced the number of CD68 and iNOS-positive cells and concurrently increased the number of CD206-positive cells, demonstrating the strongest capacity to shift synovial macrophages from an M1 to an M2 phenotype. To understand how Met-F127-DA-HRP hydrogel induced inflammatory modulation and indirectly alleviated pain, the expressions of TNF-α and pain sensitizers, including NGF and CGRP, were investigated (Figures 6A and S16A). The Met-F127-DA-HRP hydrogel effectively downregulated TNF-α, NGF, and CGRP expression in the OA synovium compared to other treatments (Figure 6E–G), which suggests that it not only modulated inflammatory responses but also inhibited nociception and pain severity and consequently, delayed the progression of OA.

**Figure 6.**
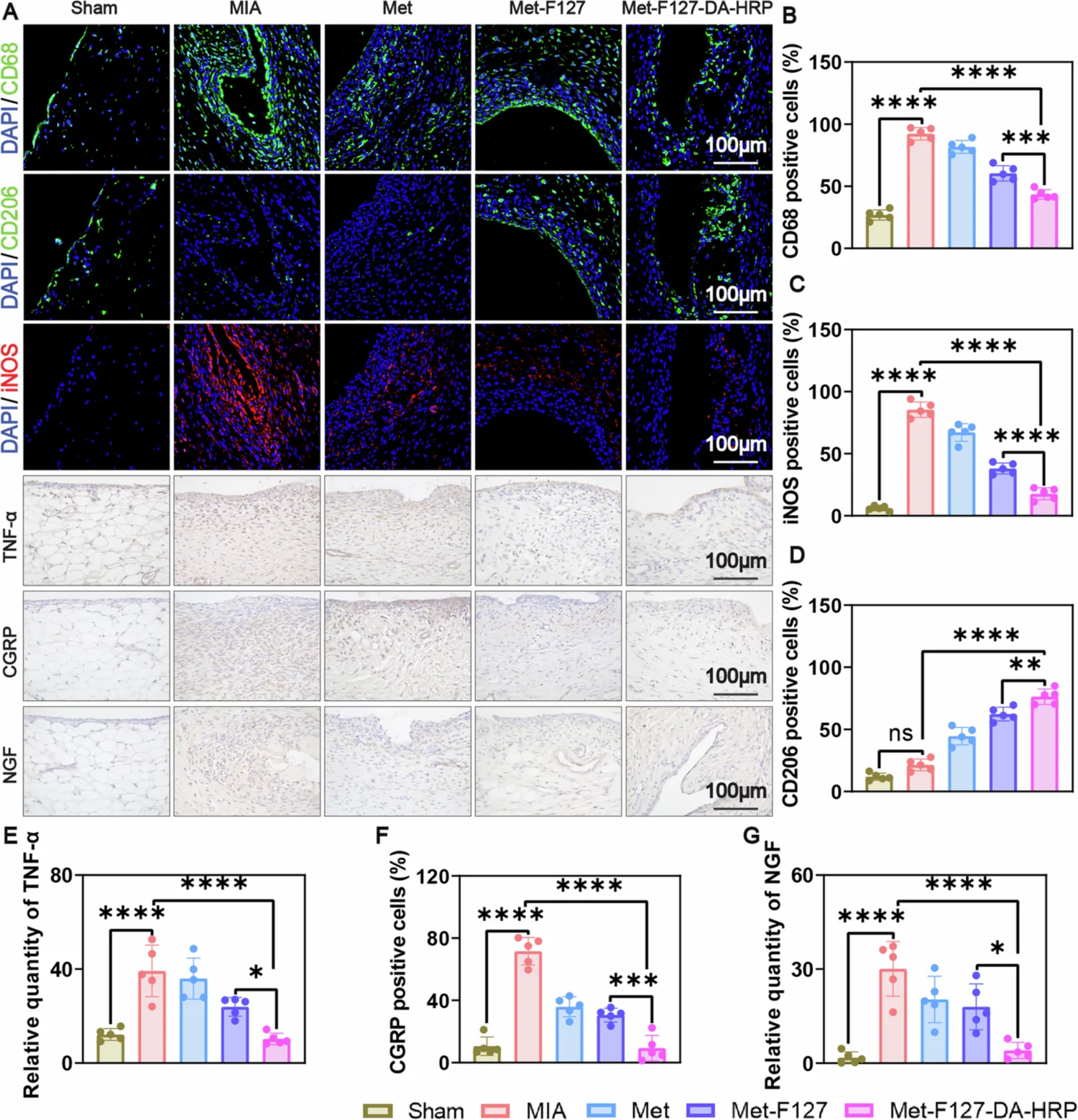
Met-F127-DA-HRP hydrogel reduces synovial M1 polarization and modulates inflammatory and pain sensitizers in the joints of MIA rats. (A) Representative images of immunofluorescence and immunohistochemistry staining for CD68, CD206, iNOS, TNF-α, CGRP, and NGF of joints from MIA rats receiving indicated treatments. (B–G) Quantitative analysis of the levels of CD68, iNOS, CD206, TNF-α, CGRP, and NGF (*n* = 5). All data represent as the mean ± S.D. *p < 0.05, **p < 0.01, ***p < 0.001, and ****p < 0.0001.

## 4. Discussion

In this study, we developed a novel injectable hydrogel system, Met-F127-DA-HRP, for OA therapy. The synthesized F127-DA-HRP hydrogel exhibited excellent ROS-eliminating properties and achieved long-term sustained drug release with a rapid gelling process. Notably, a single IA injection of Met-F127-DA-HRP hydrogel significantly attenuated pain behavior and suppressed synovial inflammation in OA models. The sustained anti-inflammatory effects of the Met-F127-DA-HRP hydrogel were attributed to its ability to inhibit the activation of M1 macrophages while inducing their transformation to M2 macrophages.

Hydrogel delivery systems have been widely employed in OA research to encapsulate conventional anti-inflammatory drugs (e.g. celecoxib (Cokelaere et al. 2018), meloxicam (Cokelaere et al. 2018; Fattahpour et al. 2020), and dexamethasone (Wang et al. 2021). Loading these drugs into hydrogels has been shown to extend release kinetics and potentiate their anti-inflammatory efficacy relative to conventional formulations. As an injectable IA delivery system, the F127-DA-HRP hydrogel exhibits reverse thermo-gelling behavior, transitioning to a gel state at physiological temperature while remaining flowable at ambient conditions. Although the reverse thermo-gelling behavior has been widely exploited in injectable systems such as the HA-Pluronic F-127 hydrogel reported by Song et al. (Song et al. 2024), our F127-DA-HRP system exhibits a markedly lower gelation temperature (22.7 °C). This lower transition point facilitates rapid sol-gel transition upon exposure to physiological temperature, thereby ensuring robust retention at the local administration site and minimizing premature diffusion. The use of H₂O₂ as a strong oxidant initiated the rapid oxidation of a portion of dopamine to dopamine-quinone, which subsequently mediated intermolecular crosslinking, thereby enhancing the cohesive strength of the polymer network. The enzymatic crosslinking effectively ‘locks’ the micellar structure, thereby restricting the diffusion pathways for encapsulated drugs, which is a critical improvement over the dissolution-dependent release observed in physical F127 gels. This oxidation reaction concurrently reduced H₂O₂ to water, a non-toxic byproduct, contributing to the biocompatibility of the curing process (Tran et al. 2010). In our study, the elastic modulus of the F127-DA-HRP hydrogel was 1.7 times greater than that of the F127 hydrogel at 37 °C (Figure 1E). Moreover, the shear modulus of the F127-DA-HRP hydrogel remained constant over a wide range of strains. All of the above properties are conducive to the stability of the hydrogel in a frequently moving joint cavity, supporting its potential for long-term therapeutic application.

Accumulating clinical evidence indicates that synovitis, a prevalent pathological feature in OA patients, is frequently associated with elevated M1 macrophage infiltration and activation in the synovium, leading to excessive ROS production (Chen et al. 2019; Yu et al. 2024). This oxidative burden disrupts redox homeostasis, drives mitochondrial dysfunction, and sustains synovial inflammation (Zhou et al. 2019). Notably, the M1-to-M2 phenotypic switch in macrophages is governed by mitochondrial metabolic reprogramming, wherein scavenging mitochondrial reactive oxygen species (mtROS) and inhibiting mitochondrial nitric oxide synthase (mtNOS) redirect cellular metabolism from glycolysis toward oxidative phosphorylation (Zhang et al. 2022). Given this, the ROS-scavenging functionality of the present catechol-functionalized hydrogel system is particularly relevant. The introduction of H₂O₂ or AAPH to the F127-DA-HRP hydrogel induced a characteristic absorption peak at 375 nm, confirming that the hydrogel's ROS scavenging capability was mediated by unoxidized catechol groups (Figure 2A). The enhanced metformin release kinetics (Figure 2B) and structural swelling of the hydrogel (Figure 2C) were mechanistically linked to ROS-induced oxidative cleavage of polyphenol crosslinks (Pu et al. 2024). This process increases network porosity, facilitating drug diffusion. Moreover, as shown in Figure 2C, the responsive mechanism of the F127-DA-HRP hydrogel was initiated on the surface, where ROS induces the formation of irregular pores. This response then propagates inward as the pores progressively penetrate the hydrogel matrix (Xu et al. 2016). What's more, we observed that the intracellular ROS scavenging capacity in the Met group was weaker than that in the F127-DA-HRP hydrogel group (Figure 2F). Previous reports have indicated metformin's concentration-dependent inhibition of mitochondrial function (Feng et al. 2022), potentially explaining its limited ROS-scavenging efficacy in the Met group. This mitochondrial inhibition may synergize with catechol-mediated ROS scavenging: by simultaneously attenuating oxidative stress and modulating mitochondrial activity, the combination creates a metabolic environment conducive to oxidative phosphorylation and, consequently, M2 repolarization (Li et al. 2024). This synergy was evidenced by the Met-F127-DA-HRP hydrogel, which demonstrated higher efficiency in repolarizing M1 macrophages compared to the Met and F127-DA-HRP hydrogel groups (Figure 3A, B). The M1 and M2 related mRNA expression levels and protein expression presented the same above trend (Figure 3C–N).

In this study, we focused on improving metformin's therapeutic efficacy by addressing its rapid clearance and degradation *in vivo*. Pharmacokinetic analysis and fluorescence imaging confirmed that a single IA injection of Met-F127-DA-HRP hydrogel significantly prolonged metformin retention time in the joint for up to 30 days (Figure 4E–G). Although thermosensitive hydrogels have been widely investigated for IA delivery, their translational potential is often hampered by an unsatisfactory retention lifespan, typically less than 3 weeks (Zhang et al. 2024; Ge et al. 2025; Zhang et al. 2025; Liao et al. 2025). Notably, our F127-DA-HRP system achieved extended retention beyond this typical timeframe, primarily attributed to its mechanically robust network and sustained release profile. This prolonged retention, together with the sustained drug release kinetics, supports a single-injection strategy as a viable approach to achieving sustained therapeutic efficacy with reduced administration frequency. Our previous study found that the administration of metformin twice weekly by IA injection can effectively relieve pain in OA models (Jin et al. 2020). Strikingly, in contrast to this frequent dosing regimen, a single injection of either the Met-F127 or Met-F127-DA-HRP hydrogel significantly attenuated synovial macrophage infiltration and M1 macrophage polarization (Figure 6A–D), thereby substantially reducing pain sensitivity and synovial inflammation in MIA-induced OA rats. Notably, the Met-F127-DA-HRP hydrogel demonstrated superior efficacy in mitigating synovitis and pain compared to the Met-F127 hydrogel. Furthermore, both the F127 and F127-DA-HRP hydrogel significantly mitigated cartilage degeneration (Figure 5G, H), as evidenced by reduced surface erosion and fibrillation, an effect primarily attributable to their biolubricating properties. IA drug delivery faces dual physicochemical barriers: penetration resistance imposed by anionic dense ECM-mediated and rapid clearance via the articular lymphatic-capillary network. The F127-DA-HRP hydrogel, after ROS oxidation, loses its ability to interact with cell surfaces and penetrate the extracellular matrix to reach chondrocytes. We therefore propose that its protective effect against cartilage degeneration is primarily achieved through its bio-lubricating function, which compensates for the deficiency in synovial fluid secretion during synovitis. In addition, F127-DA-HRP hydrogel demonstrated good biosafety *in vivo*. Given that OA pathogenesis involves complex mechanical and biological interactions (Hunter and Bierma-Zeinstra 2019), conventional biolubricants primarily reduce mechanical wear but exhibit limited effects on cellular homeostasis. In this study, the Met-F127-DA hydrogel represents a multifunctional therapeutic strategy by combining biolubrication with regulating macrophage polarization and ROS scavenging, thereby addressing both mechanical and biological aspects of OA pathology.

In this study, several limitations must be acknowledged. First, the *in vivo* ROS-scavenging activity of the Met-F127-DA-HRP hydrogel was not directly measured. While *in vitro* experiments confirmed its antioxidant capacity, and the observed suppression of synovitis and macrophage repolarization is consistent with an ROS-mediated mechanism, direct measurement of intra-articular ROS levels would be needed to conclusively establish this causal link *in vivo*. Such measurements should be incorporated in future studies to strengthen the mechanistic understanding of this system. Additionally, the F127-DA-HRP hydrogel exhibited weak tissue adhesion, which may compromise its long-term retention in the joint. Although the hydrogel demonstrated sustained metformin release for up to 30 days *in vitro*, the lack of robust adhesion to articular cartilage or synovial tissues could compromise its *in situ* residence time under mechanical loading and synovial fluid turnover. Future optimization of the crosslinking conditions to balance network formation with preservation of free catechol groups may improve tissue adhesion. These limitations highlight important directions for further optimization and preclinical evaluation before clinical translation.

## 5. Conclusion

In this study, we successfully developed an injectable Met-F127-DA-HRP hydrogel with ROS-scavenging capabilities, along with extended IA retention. The hydrogel scavenges ROS through its catechol groups and sustained metformin release, while also promoting M1-to-M2 macrophage repolarization and suppressing inflammatory cytokine secretion. Consequently, compared to an equivalent dose of free drug, the Met-F127-DA-HRP hydrogel demonstrated superior and longer-lasting efficacy in reducing pain and alleviating inflammation, thereby attenuating OA progression without observed cytotoxicity. Therefore, the injectable Met-F127-DA-HRP hydrogel provides a multifunctional and clinically translatable platform for minimally invasive treatment of OA.

## Supplementary Material

Supplementary Material Clean copy of _Supplementary_Materials UDRD-2026-0081.R1.docx

## Data Availability

All data supporting the conclusions of this paper are available within the main text or the Supplementary Materials. The data that support the findings of this study are available from the corresponding author upon reasonable request.
