# Supplementary material for: Sustained release of metformin through an injectable hydrogel for synergistic macrophage reprogramming and ROS scavenging in the analgesic treatment of osteoarthritis
Source: Drug Deliv. 2026 Oct 29;33(1):2738344. doi: 10.1080/10717544.2026.2738344 (PMC13721827; doi:10.1080/10717544.2026.2738344)
Supplement: Supplementary file 1 [file IDRD_A_2738344_SM8608.docx]

**Fig. S1.** A scheme for the synthesis of F127-DA.

**Fig. S2.** FTIR spectra of F127-NPC and F127-DA.

**Fig. S3.** ^1^H NMR spectra of F127-NPC and F127-DA (400 MHz, CDCl_3_).

**Fig. S4.** Characterizations of a series of F127-DA-HRP hydrogels. (A) SEM images showing the cell adhesion on 0.5%F127-DA-HRP, 1%F127-DA-HRP and 2% F127-DA-HRP hydrogels. (B) Storage moduli (G′) and loss moduli (G″) of 0.5%F127-DA-HRP, 1%F127-DA-HRP and 2% F127-DA-HRP hydrogels as a function of temperature from 10 to 40 °C. (C) The max storage moduli of 0.5%F127-DA-HRP, 1%F127-DA-HRP and 2% F127-DA-HRP hydrogels (*n* = 3). (D) Axial compressive stress−strain curves of the hydrogels within an 80% compression strain. All data represent as the mean ± S.D. ****p* < 0.001.

**Table. S1.** Gelation Time and Gelation Temperature of F127 and F127-DA-HRP Hydrogel (*n* = 3).

| Hydrogel group | F127 | F127-DA-HRP |
| --- | --- | --- |
| Gelation time (s) | 53.6 ± 3 | 31.3 ± 2 |
| Gelation temperature (℃) | 32.8 | 22.7 |

**Fig. S5.** *In vitro* degradation of F127-DA-HRP Hydrogel in human OA synovial fluid (*n* = 3).

**Fig. S6.** Encapsulation efficiency of metformin in the Met-F127-DA-HRP hydrogel. (*n* = 3).

**Fig. S7.** Lubrication propertiesof the F127-DA-HRP hydrogel. (A and B) COF-time plots (A) and COF histograms (B) for PBS, F127 and F127-DA-HRP hydrogel under the loading of 1 N (*n* = 3). All data represent as the mean ± S.D. ****p* < 0.001.

**Fig. S8.** Drug release profiles of the Met-F127-DA-HRP hydrogel in PBS and 600mM AAPH (37 ◦C) for the first 96 h.

**Fig. S9.** Pore size distribution statistics of the F127-DA-HRP hydrogel treated with PBS, 600 mM AAPH, 20 mM H_2_O_2_, or 5 mM NaClO over 1 h (*n* = 6 fields per group). All data represent as the mean ± S.D. ***p* < 0.01, ****p* < 0.001.

**Fig. S10.** Relationship among ROS intensity, structural changes, and metformin release behavior of the F127‑DA‑HRP hydrogel. (A) Metformin release profiles from the F127-DA-HRP hydrogel in graded concentrations of AAPH over 120 h at 37 °C. (B) Pore size distribution statistics of the F127-DA-HRP hydrogel after exposure to graded AAPH concentrations at 24 h and 72 h. (*n* = 6 fields per group). (C) Representative SEM images of the F127-DA-HRP hydrogel treated with graded AAPH concentrations at 24 h and 72 h. All data represent as the mean ± S.D. **P* < 0.05, ***P* < 0.01, ****P* < 0.001.

**Fig. S11.** Cell viability (24 h and 48 h) of RAW264.7 cells incubated with metformin (*n* = 3). All data are present the mean ± S.D. ***p* < 0.01.

**Fig.S12.** (A) Quantitative analysis of DCFH-DA fluorescence intensity in the presence of F127-DA-HRP hydrogel, Met, Met-F127-DA-HRP hydrogel, or PBS (*n* = 3). (B) Quantitative statistics of iNOS-positive cells (*n* = 3). (C) Quantitative statistics of CD206-positive cells (*n* = 3). All data represent as the mean ± S.D. ***p* < 0.01, ****p* < 0.001, *****p* < 0.0001.

**Table. S2**. Primer sequences used for quantitative real-time PCR.

| Gene | Forward primer | Reverse primer |
| --- | --- | --- |
| *iNOS* | CAGCATGTACCCTCGGTTCT | GGGGATCTGAATGTGCTGTT |
| *IL-1β* | TGAAGTTGACGGACCCCAAA | CGGGAAAGACACAGGTAGCT |
| *IL-6* | TTCACAAGTCGGAGGCTT | CAGTTTGGTAGCA TCCAT |
| *TNF-α* | CCCCAGGGACCTCTCTCTAA | TGAGGTACAGGCCCTCTGAT |
| *Arg 1* | CCCTTTGCTGACATCCCTAA | GACTCCAAGATCAGGGTGGA |
| *IL-4* | GGTCTCAACCCCCAGCTAGT | GCCGATGATCTCTCTCAAGTGAT |
| *IL-10* | GGCAGAGAACCATGGCCCAGAA | AATCGATGACAGCGCCTCAGCC |
| Col2a1 | CACACTGGTAAGTGGGGCAAGACCG | GGATTGTGTTGTTTCAGGGTTCGGG |
| ACAN | CCTGCTACTTCATCGACCCC | AGATGCTGTTGACTCGAACCT |
| Mmp13 | ATGGCACTGCTGACATCATG | AAAAGCGTGTGCCAGAAGAC |
| Adamts5 | CCTGTTCACCCGAGAGGATT | TTTGGACCAGGGCTTGGATG |
| *β-actin* | GGCTGTATTCCCCTCCATCG | CCAGTTGGTAACAATGCCATGT |

**Fig. S13.** Relative mRNA expression levels of *Col2a1, Acan, MMP13,* and *ADAMST5* (*n* = 3). All data represent the mean ± S.D. **p* < 0.05, ***p* < 0.01, and ***p < 0.001.

**Fig. S14.** **Intra-articular injection of F127-DA-HRP hydrogel preserves cartilage structure in MIA rats.** (A) Mechanical sensitivity was measured using von Frey filaments every week in MIA rats. (B) Changes in weight distribution in rats every week after surgery. P values were compared between F127-DA-HRP (*) group and MIA group. (D and E) Changes in spontaneous activity, including rearing duration (D) and rearing frequency (E) were evaluated using the LABORAS 4 weeks after MIA injection (*n* = 5). (F) The synovitis scores of samples from the different groups (*n* = 5). (G) The OARSI scores of cartilage samples from the different groups (*n* = 5). (H) Representative images of H&E and Safranin O/Fast green-stained sections of knee joints from rats in different groups. All data represent the mean ± S.D. ***p* < 0.01, ****p* < 0.001, and *****p* < 0.0001.

**Fig. S15.** (A) TUNEL immunofluorescence staining in knee joints from rats receiving the indicated treatment. (B) Quantitative analysis for the immunofluorescence of TUNEL staining in knee joints of MIA rats after receiving the indicated treatment (*n* = 3). All data represent the mean ± S.D. ***p < 0.001.

**Fig. S16.** **Synovial M1 polarization and expression of inflammatory and pain‑related mediators in MIA rats treated with the F127‑DA‑HRP hydrogel**. (A) Representative images of immunofluorescence and immunohistochemistry staining of CD68, CD206, iNOS, TNF-α, CGRP, and NGF of joints from MIA rats receiving indicated treatments. (B-G) Quantitative analysis of CD68, iNOS, CD206, TNF-α, CGRP, and NGF (*n* = 5). All data represent the mean ± S.D. **p* < 0.05 and *****p* < 0.0001.
